# Nucleoside Analogue Reverse Transcriptase Inhibitors Improve Clinical Outcome in Transcriptional Active Human Parvovirus B19-Positive Patients

**DOI:** 10.3390/jcm10091928

**Published:** 2021-04-29

**Authors:** Heinz-Peter Schultheiss, Thomas Bock, Heiko Pietsch, Ganna Aleshcheva, Christian Baumeier, Friedrich Fruhwald, Felicitas Escher

**Affiliations:** 1 Institute of Cardiac Diagnostics and Therapy, 12203 Berlin, Germany; BockC@rki.de (T.B.); heiko.pietsch@ikdt.de (H.P.); ganna.aleshcheva@ikdt.de (G.A.); christian.baumeier@ikdt.de (C.B.); felicitas.escher@charite.de (F.E.); 2 Department of Cardiology, Campus Benjamin Franklin, Charité-Universitätsmedizin Berlin, 12200 Berlin, Germany; 3 Institute of Tropical Medicine, University of Tübingen, 72074 Tübingen, Germany; 4 Department of Internal Medicine and Cardiology, Campus Virchow Klinikum, Charité-Universitätsmedizin Berlin, 13353 Berlin, Germany; 5 DZHK (German Centre for Cardiovascular Research), Berlin, Germany; 6 Department of Cardiology, Medical University Graz, 8036 Graz, Austria; friedrich.fruhwald@medunigraz.at

**Keywords:** parvovirus B19, dilated inflammatory cardiomyopathy, telbivudine

## Abstract

Human parvovirus B19 (B19V) is the predominant cardiotropic virus associated with dilated inflammatory cardiomyopathy (DCMi). Transcriptionally active cardiotropic B19V infection is clinically relevant and triggers adverse long-term mortality. During the study; we evaluated whether antiviral treatment with the nucleoside analogue telbivudine (LTD) is effective in suppressing transcriptional active B19V in endomyocardial biopsies (EMBs) of B19V positive patients and improving clinical outcomes. Seventeen B19V-positive patients (13 male; mean age 45.7 ± 13.9 years; mean left ventricular ejection fraction (LVEF) 37.7 ± 13.5%) with positive B19V DNA and transcriptional activity (B19V mRNA) in EMBs were treated with 600 mg/d LTD over a period of six months. Patients underwent EMBs before and after termination of the LTD treatment. B19V RNA copy numbers remained unchanged in 3/17 patients (non-responder) and declined or disappeared completely in the remaining 14/17 patients (responder) (*p* ≤ 0.0001). Notably; LVEF improvement was more significant in patients who reduced or lost B19V RNA (responder; *p* = 0.02) in contrast to non-responders (*p* = 0.7). In parallel; responder patients displayed statistically significant improvement in quality of life (QoL) questionnaires (*p* = 0.03) and dyspnea on exertion (*p* = 0.0006), reflecting an improvement in New York Heart Association (NYHA) Classification (*p* = 0.001). Our findings demonstrated for the first time that suppression of B19V transcriptional activity by LTD treatment improved hemodynamic and clinical outcome significantly. Thus; the present study substantiates the clinical relevance of detecting B19V transcriptional activity of the myocardium.

## 1. Introduction

Virus-induced inflammatory cardiomyopathy (DCMi) represents a major cause of heart failure with potential for transition to the clinical picture of dilated cardiomyopathy (DCM). Human parvovirus B19 (B19V) is the predominant cardiotropic virus found in DCM hearts and chronic myocarditis [1,2,3,4,5,6]. Whereas latent B19V infection has presumably no effect on the course of DCMi [7,8], it was shown that transcriptionally active B19V leads to an altered cardiac gene expression in EMB. Furthermore, replicative B19V in DCMi is an unfavorable prognostic trigger of adverse mortality [9].

B19V is a member of the genus Erythroparvovirus of the family *Parvoviridae* harboring a linear, single-stranded DNA-genome of 5.6 kb that codes for the non-structural protein NS1 and two structural capsid proteins, VP1 and VP2. Viral gene and protein expression are controlled by a combination of alternative splicing and internal polyadenylation [10,11,12,13]. Based on its strong erythroid tropism and related acute disease association, it has been shown that B19V is infecting erythroid endothelial progenitor cells and causes endothelial dysfunction in myocardial tissue [14,15,16]. Moreover, the presence of B19V remains a single independent predictor for reduced cardiac capillary density in patients with cardiomyopathy. These results demonstrate the causality between B19V infection and reduced coronary blood flow leading to endothelial dysfunction and ischemia. This is an explanation for why acute endothelial cell B19V-infection in myocarditis is associated with a cardiac microvascular impairment mimicking myocardial infarction [17,18,19].

To date, therapeutic options against B19V infection have not yet been established. The first beneficial effects following telbivudine (LTD) treatment in B19V-associated DCMi were described in a case report of EMB analyses of our group, owing not only to its antiviral but presumably also to its immunomodulatory properties [20]. This drug is approved for the treatment of hepatitis B virus (HBV) infection [21]. LTD is a synthetic thymidine β-L-nucleoside analogue and impairs HBV DNA replication by incorporation into the HBV DNA intermediate by the HBV polymerase, competing with the natural substrate thymidine-50-triphosphate. This finally leads to chain termination by interruption of the HBV second strand synthesis [22,23]. Additionally, LTD shows a broad range of immune modulatory effects altering the expression of cytokines, such as tumor necrosis factor -(TNF-) α and interferon- (IFN)-γ, influences NF-κB level restoring cellular immune response, and reveals a direct effect on apoptosis [24,25,26,27].

In this observation, we retrospectively analyzed whether treatment with telbivudine is effective in suppressing B19V replicative activity in the myocardium and improving clinical outcomes.

## 2. Materials and Methods

### 2.1. Patients

Patients with clinical evidence of symptomatic heart failure of unknown cause and suspected inflammatory/viral cardiomyopathy underwent EMB after invasive exclusion of coronary artery disease by left heart catheterization. Indication of EMB was based on the position statement of the *ESC Working Group on Myocardial and Pericardial Diseases* [28]. Other clinical exclusion criteria were valvular disease, obstructive or restrictive cardiomyopathy, stroke, significant hepatic, renal, pulmonary, or endocrine disease, pregnancy or lactation, antiviral, immunomodulatory, or immunosuppressive therapy within six months prior to enrolment.

Patients complained of cardiac discomfort (e.g., angina pectoris at rest), atypical fatigue, and symptomatic heart failure (reduced physical capacity, dyspnea on exertion) for more than 3 months before first presentation in the clinics.

**Figure 1:**
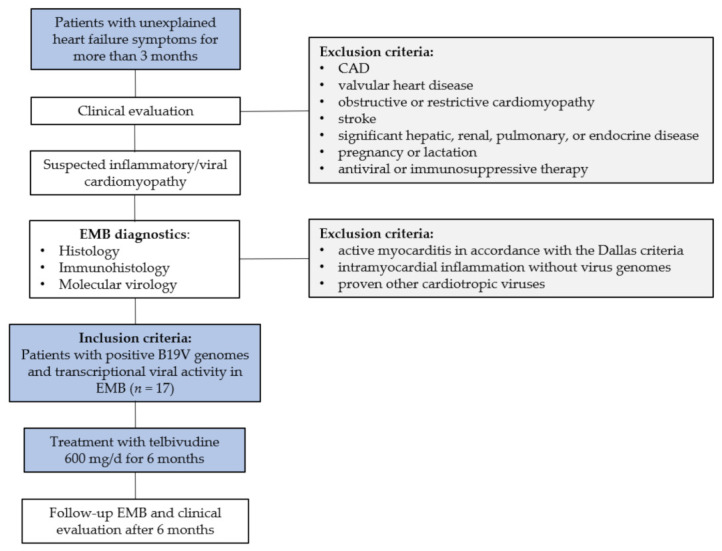
Study treatment scheme of patients with B19V transcriptional activity in EMB. B19V = parvovirus B19; CAD = coronary artery disease; EMB = endomyocardial biopsy.

EMBs were analyzed using histology, immunohistology, and molecular virology. The inclusion criterion for this study was B19V positivity with transcriptional virus activity (viral mRNA) in EMBs. Exclusion criteria were active myocarditis in accordance with the Dallas classification [29], inflammatory cardiomyopathy without viral genomes, or other cardiotropic virus infections in EMB (Figure 1).

Seventeen patients (13 male; mean age 45.7 ± 13.9 years) with different baseline left ventricular ejection fraction (LVEF) (mean LVEF 37.7 ± 13.5; range [26.5–51.0]%), fitting the criteria of this study with positive B19V DNA and replicative intermediates (positive B19V mRNA) in EMBs, were included (Figure 1). The present study retrospectively investigates data from a prospective observational cohort from 1 single center, which neither includes randomization or matching of patients nor does it comprise a control group.

Patients were treated with 600 mg/d LTD over a period of six months. Patients underwent EMBs before (pre/baseline) and immediately after termination (post/follow-up) of the LTD treatment. The clinical status of each patient was re-evaluated at follow-up after six months. A determination of LVEF was performed by echocardiography. Patients were examined with 2-dimensional echocardiography using the Philips iE33 ultrasound system (Philips Healthcare, Germany). LVEF measurements were performed with the Simpson method.

Initially, health insurance companies were informed from the clinicians and gave consent in “off-label” use reimbursement.

The analysis was performed within the CRC Transregio 19 (NCT02970227), which was approved by the local ethics committees of the participating clinical centers as well as by the committees of the respective federal states. Informed written consent was obtained from all patients.

### 2.2. Analysis of EMB

#### 2.2.1. Genomic DNA Isolation from EMBs

EMBs were analyzed in the CAP-accredited laboratory Institute for Cardiac Diagnostic and Therapy Berlin, Germany (IKDT) by molecular workup: Genomic DNA from RNAlater (Thermo Fisher Scientific, Waltham, MA, USA) fixed EMBs were extracted by Gentra Puregene Mousetail Kit (Qiagen, Hilden, Germany). After isolation, the amount of DNA was quantified by Quantifiler™ Human DNA TaqMan assay (Thermo Fisher Scientific, Waltham, MA, USA), in order to calculate and standardize viral load in small EMBs (viral genomes per µg of isolated human genomic DNA) [30].

#### 2.2.2. Detection of Viral Genomes in EMBs by Nested-PCR and Sequencing

Nested polymerase chain reaction (nested-PCR) and reverse transcriptase (RT)-PCR for qualitative detection of B19V, enteroviruses (including coxsackieviruses and echoviruses), adenoviruses, Epstein–Barr virus, and human herpesvirus 6 genome sequences in nucleic acids were extracted from EMB, performed as described previously [30].

The specificity of PCR products was confirmed by DNA sequencing and sequences were matched to the NCBI GenBank. DNA sequence analysis for quality control and genotype/species determination of generated nested-PCR amplicons was performed by PCR using corresponding primer pairs matching the amplified virus fragment.

#### 2.2.3. Measurement of Viral DNA Load by Quantitative Real-Time PCR (TaqMan qPCR)

Subsequently, calculation of viral DNA load was performed by the ratio of viral genome copy number in TaqMan assay to the amount of isolated total human DNA measured by Quantifiler™ Human DNA TaqMan assay (Thermo Fisher Scientific, Waltham, MA, USA) [9].

#### 2.2.4. RNA Isolation, Reverse Transcription (RT), and TaqMan qPCR for Measurement of Viral Transcripts

Total RNA was isolated from endomyocardial biopsies using TRIzol reagent (Thermo Fisher Scientific, Waltham, MA, USA), treated with DNAse (PeqLab, Erlangen, Germany) to remove any traces of DNA and reverse-transcribed to cDNA with the High-Capacity cDNA Reverse Transcription Kit (Applied Biosystems, Darmstadt, Germany) using random hexamers.

The amount of viral transcripts in cDNA was determined by real-time PCR using TaqMan Universal PCR master mix (Thermo Fisher Scientific, Waltham, MA, USA) in relation to housekeeping-gene HPRT (viral transcripts per µg of isolated human genomic RNA) (Applied Biosystems, Darmstadt, Germany) as described previously [31].

#### 2.2.5. Histological and Immunohistochemical Staining for Assessment of Inflammation

Histology was developed by hematoxylin eosin staining in light microscopy. Myocardial inflammation was diagnosed by CD3^+^ T-lymphocytes/mm^2^ (Dako, Glostrup, Denmark), CD11a^+^/LFA-1^+^ lymphocytes/mm^2^ (Immuno Tools, Friesoythe, Germany), CD11b^+^/Mac-1^+^ macrophages/mm^2^ (ImmunoTools, Friesoythe, Germany), and CD45R0^+^ T memory cells/mm^2^ (Dako, Glostrup, Denmark). Stainings were quantified by digital image analysis as described previously [32]. Intramyocardial inflammation was assigned by >14 leucocytes [28]. Furthermore, we analyzed macrophages (threshold > 40.0 CD11b^+^/Mac-1^+^ macrophages/mm^2^) and CD45R0^+^ T Memory cells (threshold > 40 cells/mm^2^).

### 2.3. Statistics

Qualitative data were compared using the Fisher’s exact or χ2 test. Shapiro–Wilk normality tests were used to test for normal distribution of data. When data showed normal distribution, a two-tailed parametric paired *t*-test was used to analyze continuous variables; otherwise, Wilcoxon matched-pairs signed rank test was applied. Results for quantitative features are given as mean ± SD or median values. *p*-values below 0.05 were considered to indicate statistical significance. All statistical analyses were performed using the SPSS software version 23.0 (IBM Corp., Armonk, NY, USA) and GraphPad Prism 7.04 software (GraphPad Software Inc., La Jolla, CA, USA).

## 3. Results

Patients included in this analysis were in clinically stable condition with an impaired systolic mean LVEF of 37.7 ± 13.5 [range 26.5–51.0]% (Table 1). There was a history of infection preceding onset of symptoms < 12 weeks in 70.1% of patients (Table 1). Complaints included mostly dyspnea on exertion (88.2%), reflected in New York Heart Association (NYHA) class II/III (Table 1). Clinical data of the total patient cohort at baseline are summarized in Table 1.

Patients with active myocarditis in accordance with the Dallas criteria or viral co-infections in EMB were excluded.

LTD treatment was started immediately after evaluation of baseline EMB. Interruptions in study medication were not reported.

### 3.1. EMB Analyses at Baseline and Follow-Up

At baseline, EMBs of all patients tested positive for presence of B19V DNA and viral replicative intermediates (B19V mRNA).

**Figure 2:**
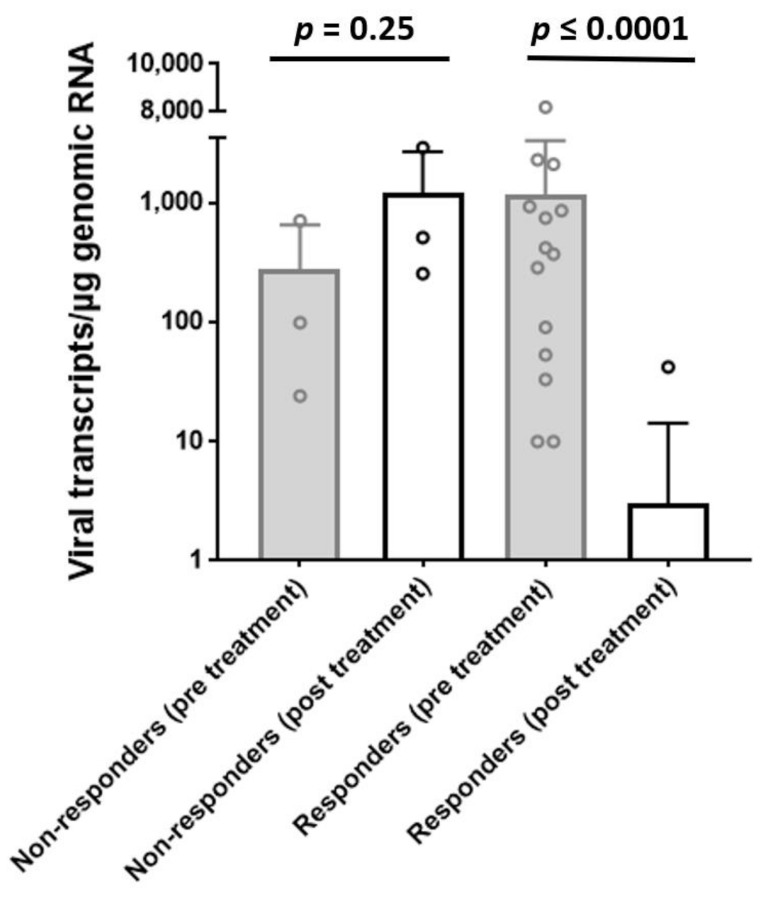
Number of viral RNA transcripts of non-responders and responders pre- and post-treatment. Bar height indicates the mean value ± SD expression rate of viral transcripts/µg RNA in non-responders’ (*n* = 3) and responders’ group (*n* = 14) pre (baseline) and post (follow-up) LTD-treatment. Dots indicate individual values. Whereas responders significantly reduce viral replication intermediates upon treatment, non-responders show a non-significant increase of viral RNA. Viral transcripts are given as mean value and error bars represent SD.

B19V RNA transcript numbers remained unchanged or increased in 3/17 patients (17.6% non-responder) and declined or disappeared completely in the remaining 14/17 patients (82.4%, responder) (mean viral RNA transcript numbers declined after treatment at follow-up in the responder group from 1168 ± 2142 to 3.0 ± 11.2 transcripts/µgRNA, *p* ≤ 0.0001 (Figure 2)). In addition, a significant reduction of viral DNA load was observed for the responders (from 1514 ± 2383 to 539 ± 1033 copies/µg DNA; *p* = 0.035) in contrast to non-responders (from 561 ± 499 to 1768 ± 2878 copies/µg DNA; *p* = 0.478) pre- and post-treatment.

**Figure 3:**
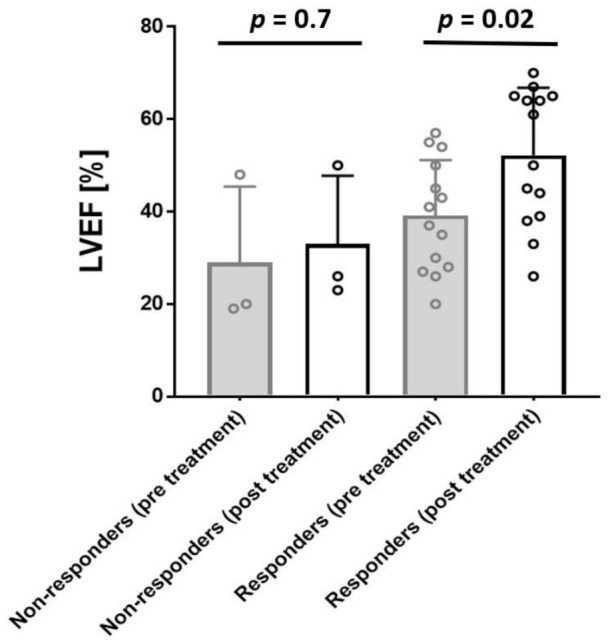
LVEF of non-responders and responders pre- and post-treatment. Bar height indicates LVEF (%) in non-responders’ (*n* = 3) and responders’ (*n* = 14) group pre (baseline) and post (follow-up) LTD-treatment. Dots indicate individual values. LVEF improvement was significantly improved in patients who reduced or lost the replicative viral intermediates (positive B19V RNA). LVEF is given as mean value and error bars represent SD.

LVEF improvement was statistically significant in patients who reduced or resolved the replicative viral intermediates (LVEF from 39.6 ± 12.4 [range 27.7–52.5] %, to 52.9 ± 15.7 [range 38.7–65.0] %, (responder, *p* = 0.02)) (Figure 3). No significant improvement of LVEF was observed in the non-responder group from 29 ± 18.2 [range 19.0–48.0] % to 35 ± 21.2% [range 23.0–50.0] %, (*p* = 0.7) (Figure 3).

Six-Minute Walk Distance (6MWD) results and quality of live (QOL) score by the Minnesota Living with Heart Failure Questionnaire (MLHFQ) before and after the treatment were assessed. Patients showed a statistically significant improvement in maximal 6MWD in responders (465.9 ± 82.8 vs. 559.8 ± 71.7 m, *p* = 0.04) and QOL total score (66.6 ± 17.7 vs. 43.2 ± 23.9; *p* = 0.03) after treatment (Table 2). In addition, a significant reduction of dyspnea on exertion (*p* = 0.0006), reflecting an improvement and New York Heart Association (NYHA) Classification (*p* = 0.001), was noticed (Table 2). No significant improvements in clinical symptoms were observed in non-responders (Table 2).

The number of inflammatory cells (CD3^+^ T-lymphocytes, CD11a^+^/LFA-1^+^ lymphocytes, CD11b^+^/Mac-1^+^ macrophages, CD45R0^+^ T memory cells) was low at baseline EMB and did not change significantly upon LTD treatment, with no difference between responder and non-responder group (Table 3).

### 3.2. LTD Side Effects

LTD antiviral treatment was well tolerated, and the majority of patients reported no drug-associated side effects. One male patient experienced an asymptomatic sudden increase of creatine kinase from <150 U/L to 3449 U/L at the time of the follow-up. This elevated creatine kinase level normalized within 5 days after immediate cessation of the treatment and remained at a normal level throughout the following 12 months.

## 4. Discussion

In this analysis, we demonstrated the beneficial effects from treatment with the antiviral drug LTD on hemodynamic and clinical outcomes in patients with transcriptionally active cardiotropic B19V in EMB.

B19V infection of the heart muscle and its association with DCMi is still a matter of discussion. Previous reports have shown that B19V is the predominant virus detected in EMBs of patients with suspected myocarditis and DCMi [28,33,34,35]. There is broad approval that latent B19V infection has no effect on the course of DCMi [7,8]. However, we could show previously that patients characterized by transcriptionally active cardiotropic B19V with detectable replication intermediates (viral mRNA) demonstrated an altered cardiac gene expression pattern. Furthermore, transcriptionally active B19V in DCMi is an unfavorable prognostic trigger of adverse mortality [9,36].

Recently, we demonstrated that endothelial dysfunction and clinical symptoms improved during antiviral treatment with interferon-β (IFN-β), whereas B19V viral DNA load was barely affected [37,38]. The underlying mechanisms of how IFN-β exerts such beneficial clinical effects without substantially clearing the virus are unknown. However, cell culture analyses using infected immortalized human microvascular endothelial cells (EC) have shown that IFN-β inhibits B19V reactivation and improves endothelial cell viability [38].

The first experimental data have provided evidence that antiviral nucleoside analogue reverse transcriptase inhibitors, such as LTD, may improve the viability of B19V infected endothelial cells in addition to their effectiveness in retroviral and para-retroviral (hepatitis B viruses) infections [39,40,41] and reduce apoptosis [20]. An advantage of the thymidine β-L-nucleoside analogue LTD is a significantly lower frequency of drug-specific side effects in comparison to IFN-β. The antiviral efficacy of LTD on non-hepatic infection is, however, less well documented [42,43,44].

Although this antiviral nucleoside analogue reverse transcriptase inhibitor is especially effective against retroviral and para-retroviral infections, it has some pleiotropic immunomodulatory/anti-inflammatory and interesting antiviral properties that might interfere with the unique replication mode of B19V. Since LTD modulates the innate and adaptive immune system through IFN-like functions in addition to its antiviral potential [45,46,47], we analyzed whether this drug may have an impact on the clinical outcome of patients with active B19V infection and persisting symptomatic heart failure during this present study, independently of baseline LVEF (Figure 1).

We could show that LTD treatment resulted in a significant amelioration of LVEF. B19V RNA transcript numbers remained unchanged in 3 of 17 patients (non-responder) and declined or disappeared completely in the remaining 14 of 17 patients (responder) (Figure 2). LVEF improvement was statistically significant in patients who reduced or lost B19V RNA (responder, *p* = 0.02) in contrast to non-responders (Figure 3). Although B19V DNA genomes in the myocardium alone are not of significant clinical relevance [7,8,34], we also observed a significant reduction of viral DNA in the responders group in contrast to non-responders.

In parallel, LTD treatment resulted in a significant amelioration of LVEF and a significant reduction of dyspnea on exertion, reflected in an improvement of NYHA class (Table 2).

The mode of action of the antiviral treatment of B19V with LTD is speculative. Clinical improvement may be associated with consecutive improvements of myocardial function or as a result of regeneration of B19V-associated endothelial dysfunction [38]. In addition, LTD shows pleiotropic immunomodulatory/anti-inflammatory and anti-viral properties that are hypothesized to interfere with the unique replication mode of B19V [48]. Since the number of inflammatory cells was low at baseline EMB, we were not able to investigate any anti-inflammatory effects upon evaluation of follow-up EMB. B19V uses the cellular DNA replication machinery for viral DNA replication by a rolling circle mechanism and induces DNA damage response and cell cycle arrest at late S phase, which facilitates viral DNA replication. DNA replication polymerase δ and polymerase α are responsible for B19V DNA replication [49]. Here, a nucleoside analogue can hypothetically interfere with the production of the obligatory dimer duplex replicative intermediate of B19V resembling second-strand synthesis of HBV DNA intermediates [50]; however, this hypothesis has to be proven in future experiments.

In a previous study by Hazebroek et al., intravenous immunoglobulin therapy does not significantly improve LVEF or functional capacity in patients with cardiac B19V persistence. However, inclusion criteria were solely based on B19 viral DNA load [51]. These results underline the importance of diagnostic differentiation of endomyocardial B19V infections (including viral replication) as a key approach for a more meaningful selection of candidates for future innovative anti-viral immunomodulatory treatment strategies.

## 5. Conclusions

In conclusion, the present study demonstrates the benefit of nucleoside-analogue treatment with respect to control of B19V replication and virus transcript reduction as well as the rapid improvement of symptoms of patients with active B19V infection. To further explore the direct and important clinical impact of our findings, a large randomized, placebo-controlled clinical study should evaluate the results and will be able to gain more insights into effective B19V treatment conditions.

## 6. Study Limitations

There are several limitations which have to be considered when interpreting the obtained results. The cohort was only adjusted to consecutive B19V-positive symptomatic patients with detectable replicative intermediates of B19V confirmed by measurement of B19V RNA transcripts. Findings do not include randomization or matching of patients nor evaluation of a placebo control. This is a retrospective analysis of data from a prospective observational cohort and, as such, a possible effect of selection bias cannot be denied.

## Figures and Tables

**Table 1:** Baseline characteristics of patients.

**Patient Characteristics**	
Men, n (%)	13 (65)
Age, years, mean ± SD	45.7 ± 13.9
History, weeks ± SD	18.9 ± 11.4
LVEF, % ± SD; [range 25–75]	37.7 ± 13.5 [26.5–51.0]
Systolic blood pressure, mmHg ± SD	102 ± 27
Diastolic blood pressure, mmHg ± SD	69 ± 8
Infection preceding onset of symptoms < 12 weeks, %	70.7
**Complaints at Baseline Biopsy**	
MLHFQ total score, mean ± SD	68.1 ± 17.7
6MWD, m, mean ± SD	468.8 ± 73.3
Dyspnea on exertion, n (%)	15 (88.2)
NYHA class I/II/III/IV, %	0/47.0/47.0/5.9
**Heart Failure Medication**	
ACE inhibitors/Angiotensin receptor blockers, n (%)	17 (100)
Beta-blockers, n (%)	15 (88.2)
Aldosterone-antagonists, n (%)	13 (76.4)
Diuretics, n (%)	16 (94.1)

LVEF = left ventricular ejection fraction; MLHFQ = Minnesota Living with Heart Failure Questionnaire. The total score ranges from 0 to 105, with higher scores indicating worse health status; 6MWD = Six-Minute Walk Distance; NYHA class = New York Heart Association class. The data are presented as mean values ± standard deviation, as %, as range [25–75 percentile], or as number of subjects.

**Table 2:** Course of clinical symptoms at baseline and at follow-up upon treatment in responders.

	Baseline	Follow-Up	*p*-Value
LVEF, % ± SD, [range 25–75]	39.6 ± 12.4 [27.7–52.5]	52.9 ± 15.7 [38.7–65.0]	*p* = 0.02
MLHFQ total score, mean ± SD	66.6 ± 16.4	43.2 ± 23.9	*p* = 0.03
6MWD, m, mean ± SD	465.9 ± 82.8	559.8 ± 71.7	*p* = 0.04
Dyspnea on exertion, n (%)	12 (85.7)	4 (28.5)	*p* = 0.0006
NYHA class I/II/III/IV, %	0/57.1/35.7/7.1	35.7/64.2/0/0	*p* = 0.001

LVEF = left ventricular ejection fraction; MLHFQ = Minnesota Living with Heart Failure Questionnaire. The total score ranges from 0–105, with higher scores indicating worse health status; 6MWD = Six-Minute Walk Distance; NYHA class = New York Heart Association class. The data are presented as mean values ± standard deviation, as %, as range [25–75 percentile], or as number of subjects.

**Table 3:** Immunohistological biopsy findings at baseline and upon treatment at follow-up in responders.

	Baseline	Follow-Up	*p*-Value
CD3^+^ cells/mm^2^	8.7 ± 7.7	4.7 ± 4.0	0.1
LFA-1^+^ cells/mm^2^	15.9 ± 11.6	10.9 ± 8.3	0.09
CD45R0^+^ cells/mm^2^	23.5 ± 15.0	20.5 ± 17.5	0.4
MAC-1^+^ cells/mm^2^	42.9 ± 41.5	28.7 ± 17.9	0.3

CD3 = T cells; CD45R0 = T memory cells; LFA-1 = Lymphocyte function-associated antigen 1; MAC-1 = Macrophage-1 antigen. The data are presented as mean values ± SD.

## Data Availability

The data presented in this study are available on request from the corresponding author.

## Abbreviations

B19V = parvovirus B19; CAD—coronary artery disease; DCM = dilated cardiomyopathy; CD3 = T cells; CD45R0 = T memory cells; DCMi = inflammatory dilated cardiomyopathy; DNA = deoxyribonucleic acid; EMB = endomyocardial biopsy; GE = genome equivalents; HBV = hepatitis B virus; HPRT = hypoxanthine phosphoribosyltransferase 1; IFN = interferon; LFA-1 = Lymphocyte function-associated antigen 1; LTD = telbivudine; MAC-1 = Macrophage-1 antigen; NYHA classification = New York Heart Association classification; QoL = quality of life; PCR = polymerase chain reaction; RT-PCR = reverse transcription PCR; TNF-α = Tumor necrosis factor-α.
